# Evaluation of Sarcopenic Obesity in Patients with MASLD

**DOI:** 10.3390/medsci14020257

**Published:** 2026-05-15

**Authors:** Niki G. Mourelatou, Triada Bali, Magdalini Adamantou, Lampros Chrysavgis, Christos Chologkitas, Margarita Sarri, Dimitra Pavlopoulou, Georgios Schinas, Theodoros Androutsakos, Georgia Sypsa, Dimitrios S. Karagiannakis, George Papatheodoridis, Nikolaos Tentolouris, Anastasia N. Mavrogiannaki, Evangelos Cholongitas

**Affiliations:** 1 First Department of Internal Medicine, Medical School, National and Kapodistrian University of Athens, General Hospital of Athens “Laiko”, 11527 Athens, Greece; 2 Second Department of Internal Medicine and Diabetes Centre, NIMTS Hospital, 11521 Athens, Greece; 3 Department of Pathophysiology, Medical School, National and Kapodistrian University of Athens, 75 Mikras Asias Str., 11527 Athens, Greece; t_androutsakos@yahoo.gr; 4 Department of Radiology, General Hospital of Athens “Laiko”, 11527 Athens, Greece; 5 Fourth Department of Internal Medicine, School of Medicine, National and Kapodistrian University of Athens, Attiko Academic Hospital, 12462 Athens, Greece; drkaragiannakis@gmail.com; 6 First Department of Gastroenterology, Medical School, National and Kapodistrian University of Athens, General Hospital of Athens “Laiko”, 11527 Athens, Greece; 7 First Department of Propaedeutic Internal Medicine, Medical School, National and Kapodistrian University of Athens, General Hospital of Athens “Laiko”, 11527 Athens, Greece

**Keywords:** MASLD, sarcopenic obesity, dual-energy X-ray absorptiometry (DEXA), dehydroepiandrosterone sulfate (DHEAS), frailty, physical performance, short physical performance battery (SPPB), liver frailty index (LFI), sarcopenia, hormones

## Abstract

Background/Obejctives: Sarcopenic obesity (SO) has gained growing attention in metabolic dysfunction-associated steatotic liver disease (MASLD), yet data in Caucasian populations remain limited. The aim of this study was to assess the prevalence of SO using different definitions and to explore its relationship with steroid androgens, physical performance and frailty in MASLD individuals. Methods: Two hundred Caucasian patients with MASLD and available dual-energy X-ray absorptiometry (DEXA) data were evaluated. Clinical, biochemical, hormonal and elastography data were recorded, while physical performance was assessed using the Short Physical Performance Battery (SPPB) and Liver Frailty Index (LFI). Results: SO prevalence ranged from 34.5% to 76.5% depending on the definition applied (AIMSO score, body mass index, and body fat percentage-based criteria). Across definitions, SO individuals showed greater hepatic steatosis, more metabolic comorbidities and demonstrated poorer physical performance. Lower dehydroepiandrosterone sulfate (DHEAS) levels were independently associated with SO when the definition is based on total body fat percentage, and waist circumference (WC) was consistently linked to SO across all definitions. Separate analysis based on gender, confirmed that DHEAS was independently associated with SO in men, while WC represented an independent factor associated with SO in both genders. Conclusions: In conclusion, SO is common among Caucasian MASLD patients and is accompanied by metabolic, hepatic, hormonal, and functional alterations. These findings may help recognize patients at risk of SO and support more focused assessment and monitoring in clinical practice.

## 1. Introduction

Metabolic dysfunction-associated steatotic liver disease (MASLD), formerly known as non-alcoholic fatty liver disease (NAFLD), was redefined to highlight its strong association with metabolic disorders [1]. MASLD encompasses a broad spectrum of disease severity ranging from simple hepatic steatosis (metabolic dysfunction-associated steatotic liver, MASL) to metabolic dysfunction-associated steatohepatitis (MASH), fibrosis and cirrhosis [2]. Nowadays, MASLD has emerged as the primary cause of hepatic disease, with a prevalence higher than 30% in the adult population and projected to reach up to 55% by 2040 [3]. Meanwhile, among individuals with obesity, the prevalence of MASLD may exceed 75%, with approximately 7% already having advanced fibrosis [4]. The impact of MASLD extends beyond the liver and is often associated with systemic complications, including cardiovascular and renal disease, sleepapnea, extrahepatic cancers, and sarcopenia. The latter is described as a reduction in muscle mass and muscle strength, which may be accompanied by limitations in physical performance [5]. Sarcopenia affects approximately 10–16% of older adults, and it is linked to increased frailty and reduced quality of life [6]. Its prevalence in MASLD may reach up to 24% representing an independent risk factor for fibrosis and being associated with a greater risk of frailty and mortality [7,8].

The coexistence of sarcopenia and obesity is defined as sarcopenic obesity (SO), with an estimated global prevalence of around 11% among older adults [9]. Instead of a neutral simultaneous presence of these two conditions, their coexistence might be associated with higher complications and mortality rates than with sarcopenia or obesity alone [10,11]. In fact, SO presence in adults has been linked to a 24% higher all-cause mortality rate and to worse cardiovascular outcomes, compared to individuals with either condition alone or neither condition [12,13]. Although the mechanisms underlying SO have not been fully clarified, shared pathways between sarcopenia and obesity are thought to contribute to SO development, such as insulin resistance, low-grade inflammation, adipose tissue dysfunction, and hormonal disturbances [14]. Regarding the latter, disruption of the hormonal milieu, reflected by lower testosterone and dehydroepiandrosterone sulfate (DHEAS) levels has been reported with aging in both men and women, contributing to the pathogenesis of SO [15,16].

**Figure 1:**
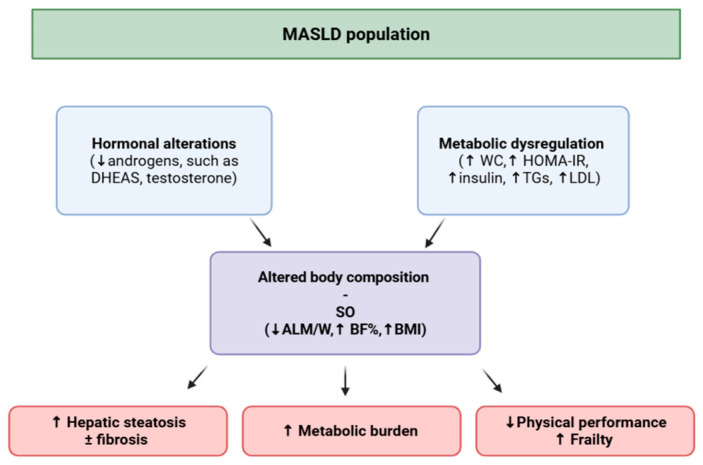
Proposed hypothetical model linking hormonal alterations (e.g., low androgens) with changes in body composition and functional performance in patients with MASLD. Abbreviations: MASLD: metabolic dysfunction-associated steatotic liver disease; DHEAS: dehydroepiandrosterone sulfate; WC: waist circumference; HOMA-IR: homeostatic model assessment of insulin resistance; TGs: triglycerides; LDL: low density lipoprotein; SO: sarcopenic obesity; ALM/W: appendicular lean mass-to-weight ratio; BF%: body fat percentage; BMI: body mass index.

In patients with MASLD, the prevalence of SO may rise to 47% [10] but this may differ depending on the definition criteria used. Nevertheless, epidemiological data on SO remains limited in MASLD, particularly in the Caucasian population [17]. In addition, although evidence suggests that SO may be associated with more advanced stages of liver steatosis and fibrosis [18,19], these studies have several limitations including the absence of more accurate evaluation of liver steatosis, such as controlled attenuation parameter (CAP), and/or precise assessment of body fat and muscle mass with methods such as dual-energy X-ray absorptiometry (DEXA) [20]. Moreover, the impact of SO on physical performance and frailty in MASLD and the possible implication of steroid androgens remains poorly explored in the MASLD population [21]. A potential hypothetical model can therefore be proposed, in which hormonal alterations together with metabolic dysregulation may contribute to changes in body composition leading to sarcopenic obesity, which in turn is associated with reduced physical performance and subsequent frailty (Figure 1).

Thus, the aim of our study was to evaluate the prevalence of SO in Caucasian patients with MASLD, to investigate its potential correlation with the severity of liver disease and frailty and to examine, for the first time, the association of steroid androgens with SO and frailty in MASLD patients concurrently.

## 2. Materials and Methods

### 2.1. Study Design and Population

This cross-sectional study included 200 individuals with a diagnosis of MASLD, followed up at the liver steatosis outpatient clinic of “Laiko” General Hospital in Athens, Greece. The inclusion criteria were aged ≥ 18 years old with MASLD and availability of body composition data measured by DEXA. Exclusion criteria included decompensated liver cirrhosis, defined as the presence or prior history of ascites, variceal bleeding, hepatic encephalopathy, or jaundice related to liver disease, acute infection confirmed clinically and by laboratory tests, hemodynamic instability, pregnancy or lactation, acquired immunodeficiency syndrome and the administration of any medication containing steroids (e.g., tablets, inhalers, or ointments, etc.) or hormone replacement therapy.

In our study population, to diagnose MASLD, the presence of hepatic steatosis along with at least one cardiometabolic risk factor was required [2]. Hepatic steatosis was identified by liver ultrasound. Cardiometabolic risk factors for MASLD definition adapted to the Greek (European) population were as follows: (i) overweight or obesity: BMI ≥ 25 kg/m^2^ or WC ≥ 94 cm in men and ≥80 cm in women; (ii) prediabetes or type 2 diabetes (T2D): hemoglobin A1c (HbA_1c_, %) 5.7–6.4%, fasting plasma glucose 100–125 mg/dL or 2 h plasma glucose during oral glucose tolerance test (OGTT) 140–199 mg/dL (prediabetes) or HbA_1c_ ≥ 6.5%, fasting plasma glucose ≥ 126 mg/dL or 2 h plasma glucose during OGTT ≥ 200 mg/dL (T2D) or assumption of antidiabetic medication; (iii) hypertrygliceridemia: triglycerides ≥ 150 mg/dL or lipid-lowering treatment; (iv) low high-density lipoprotein cholesterol (HDL cholesterol): HDL cholesterol ≤ 39 mg/dL in men and ≤50 mg/dL in women or lipid-lowering treatment; and (v) high blood pressure: blood pressure ≥ 130/85 mmHg or antihypertensive treatment [2]. The use of medications for arterial hypertension, dyslipidemia, and type 2 diabetes was recorded. Detailed information on medication classes and the number of patients receiving each treatment is provided in Supplementary Table S1. Moreover, to confirm the diagnosis other major etiologies for hepatic steatosis, including viruses and pharmaceutical agents, were excluded, while alcohol intake—based on patient self-report and the AUDIT questionnaire at the time of evaluation—was required to be limited to less than 20 g per day for women and less than 30 g per day for men [2].

### 2.2. Clinical Data and Laboratory Findings

Medical history regarding demographic characteristics and comorbidities was recorded. Anthropometric measurements included body weight (kg) in light clothing, and waist circumference (WC, cm), while body mass index (BMI) was calculated using the formula: BMI = weight (kg)/(height (m))^2^.

The main laboratory exams recorded included HbA1c, blood glucose (Glu, mg/dL), insulin (μU/mL), homeostatic model assessment of insulin resistance (HOMA-IR) index estimated using the following formula: HOMA-IR = Fasting Insulin (µU/mL) × Fasting Glucose (mg/dL)/405 [22], total cholesterol (mg/dL), HDL cholesterol (mg/dL), low-density lipoprotein cholesterol (LDL cholesterol, mg/dL), triglycerides (mg/dL), aspartate aminotransferase (AST, U/L), alanine aminotransferase (ALT, U/L), gamma-glutamyl transferase (γ-GT, U/L), 25-hydroxy-vitamin D (25(OH)VitD, ng/mL) and ferritin (ng/mL).

Regarding the hormonal profile, follicle-stimulating hormone (FSH, mIU/mL), luteinizing hormone (LH, mIU/mL) was measured using as chemiluminescent assay (Liaison, DiaSorin, Saluggia, Italy), dehydroepiandrosterone sulfate (DHEAS, μg/dL), sex hormone-binding globulin (SHBG, nmol/L), and total serum testosterone (Testo, ng/mL) were measured with electrochemiluminescence (Combass e801 Roche, Mannheim, Germany), while free testosterone (free Testo, ng/mL) was calculated indirectly using the Vermeulen formula [23]. Finally, Δ4 androstenedione (Δ4, ng/mL), measured by radioimmunoassay (Demeditec Diagnostics GmbH, Kiel, Germany), was recorded. Both biochemical and hormonal tests have been performed in the morning, (8:00–8:30 a.m.) after overnight fasting.

### 2.3. Liver Steatosis and Fibrosis Assessment

Liver steatosis and fibrosis were assessed using ultrasound-based elastography data. Hepatic steatosis was quantified by a controlled attenuation parameter (CAP, dB/m): <238 dB/m indicated absence of steatosis (S0), 238–259 dB/m mild steatosis (S1), 260–290 dB/m moderate steatosis (S2), and ≥291 dB/m severe steatosis (S3), while liver stiffness was evaluated by a Fibroscan, with values of 6–7 kPa indicating mild fibrosis (stage F1), 7–9 kPa moderate fibrosis (stage F2), 9–12 kPa advanced fibrosis (stage F3), and >12 kPa cirrhosis (stage F4) [24,25]. Finally, fibrosis-4 index (FIB-4) was also calculated using this formula: FIB-4 = ((age [years]) × (AST [U/L]))/((platelets [10^9^/L]) × (√ALT [U/L])) [26].

### 2.4. Body Composition

Body composition was assessed using DEXA (Hologic Horizon W, software version 13.6.0.4, Hologic Inc., Marlborough, MA, USA, with standard NHANES BCA calibration), which provided measurements of total and regional lean mass, fat mass and bone mineral content. Total body fat percentage (BF%) and appendicular lean mass (ALM), which reflects the muscle quantity of the limbs, were quantified. The appendicular lean mass-to-weight ratio (ALM/W, %) was further calculated by dividing ALM by body weight.

### 2.5. Evaluation of Physical Performance and Frailty

Physical performance was assessed using the Short Physical Performance Battery (SPPB) and the Liver Frailty Index (LFI). The SPPB (with score > 10 showing good physical performance) included a balance test in three positions (side-by-side, semi-tandem, tandem), a 4 m walk to assess gait speed and a chair stand test (time to rise from a chair 5 times without using arms) [27]. The LFI was calculated by measuring grip strength with a dynamometer, time to complete 5 chair stands and balance in three positions, with scores classified as robust (<3.2), prefrail (3.2–4.4) and frail (≥4.5) [28].

### 2.6. Definitions of SO

The diagnosis of SO was established by the concomitant presence of both obesity and sarcopenia. Obesity was defined either by body mass index [BMI (kg/m^2^)] ≥ 30 [29] or by total body fat percentage as assessed by DEXA [BF% > 27 in men and >38 in women] [11]. Sarcopenia was defined using the ALM/W (%), calculated from DEXA, with cut-off values < 23.47% in women and <28.27% in men [11]. SO was further defined using the AIM-SO score [SO diagnosed when score > 0.420 in men and >0.515 in women], a sex-specific algorithm derived from fat mass (FM) and appendicular skeletal muscle mass (ASM) with the following formulas, as described by Azevedo et al.: AIM-SO score for men = ((5.243 + (0.124 × FM) + (0.449 × ASM))/15.95) + 0.60, AIM-SO score for women = ((6.013 + (0.133 × FM) + (−0.837 × bone mass) + (−0.914 × ASM))/24.28) + 0.62 [30].

### 2.7. Statistical Analysis

All statistical analyses were performed using IBM SPSS Statistics software (IBM Corp. IBM SPSS Statistics for Windows. Version 30.0. Armonk, NY, USA). First, the Shapiro–Wilk test was used to assess normality of distribution, with data considered normally distributed if *p* > 0.05 and non-normally distributed if *p* ≤ 0.05. Comparisons were conducted using the independent *t*-test for normally distributed continuous variables and the Mann–Whitney U test for non-normally distributed continuous variables. Categorical variables were compared using the chi-squared test. Continuous variables are presented as mean ± standard deviation (SD) when normally distributed and as median with interquartile range (IQR: 25th–75th percentile) when non-normally distributed. Categorical variables are expressed as absolute numbers and percentages (%). Multivariable logistic regression analyses were performed to explore factors independently associated with each SO phenotype. Given the exploratory nature of the study and the limited prior evidence regarding hormonal and functional correlates of SO in MASLD, a stepwise logistic regression approach was used as a variable-reduction strategy rather than as a confirmatory predictive modeling procedure. Candidate variables were selected based on both clinical/biological plausibility and univariate associations (*p* < 0.05). Variables directly involved in the definition of each SO phenotype, such as BMI, BF%, and ALM/W, were not entered into the corresponding multivariable models to avoid circularity. In addition, highly correlated variables reflecting the same clinical construct were not simultaneously included. When several related variables were significant in univariate analysis, the clinically most relevant variable was retained. The number of variables entered into each model was restricted according to the number of outcome events to reduce the risk of overfitting. For hormonal analyses, when two or more hormones from the same endocrine axis were significant in univariate analysis, additional adjusted models were performed, including clinically relevant hormones from the same axis, regardless of their univariate significance. The discriminative ability of variables retained in the multivariable models was assessed using the area under the receiver operating characteristic (ROC) curve (AUC) [31]. A *p*-value < 0.05 was considered statistically significant. Given the exploratory nature of the study and the number of comparisons performed across different SO definitions and subgroup analyses, no formal adjustment for multiple testing was applied. Therefore, *p*-values should be interpreted with caution, with emphasis placed on effect sizes, confidence intervals, and consistency of findings across analyses.

## 3. Results

### 3.1. Main Characteristics of the Population

The main demographic, clinical and laboratory characteristics of the study population are summarized in Table 1. Among the 200 participants, the mean age was 55.86 years, and the majority were women (54.5%). Regarding anthropometric parameters, the mean BMI was 31.7 kg/m^2^, BF% 43.41%, and ALM/W 23.84%. SO diagnosis was made in 51% (*n* = 102 patients) of the population when diagnosis of obesity was based on BMI (BMI-SO), in 76.5% (*n* = 153 patients) when diagnosis of obesity was based on BF% (BF–SO) and in 34.5% (*n* = 69 patients) when diagnosed based on AIM-SO score. The prevalence of the main metabolic comorbidities was 28.1% for T2D, 49.5% for dyslipidemia and 36.9% for arterial hypertension (AH). Regarding laboratory findings, these are presented in Table 1. Finally, data from elastography showed a mean CAP at 287.79 dB/m and liver stiffness at 6.95 kPa. The remaining characteristics of the sample population are presented in Table 1.

### 3.2. Main Characteristics of Patients Correlated with SO Phenotypes

#### 3.2.1. SO Diagnosed Based on BMI and ALM/W (BMI-SO)

**Figure 2:**
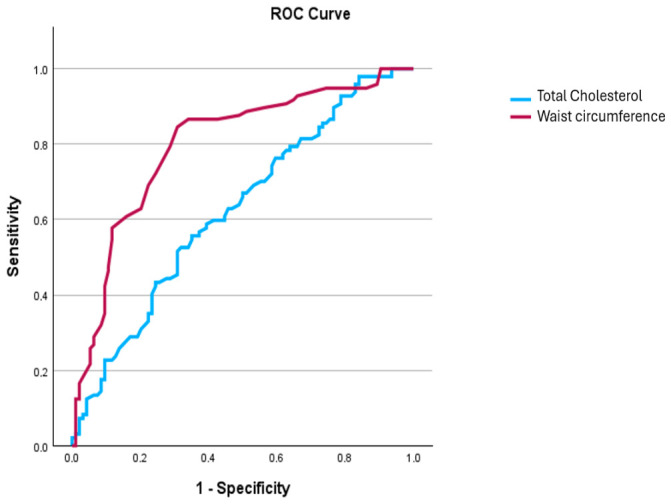
ROC curve for the discriminative ability of total cholesterol and waist circumference for the presence of body mass index-based sarcopenic obesity. Abbreviations: ROC: receiver operating characteristic.

Patients with BMI-SO, compared to those without BMI-SO, had more frequently AH and higher BMI, WC and BF%. In addition, the former group had higher levels of fasting Glu, insulin, HOMA-IR index, total cholesterol, LDL cholesterol and triglycerides. Regarding the severity of liver disease, participants with BMI-SO had worse steatosis based on CAP values (292.5 vs. 270 dB/m, *p* < 0.001), while they had poorer physical performance based on SPPB (11 vs. 12, *p* = 0.002) (Table 2). In multivariate logistic regression analysis, WC (OR: 1.085, 95% C.I.: 1.047–1.125, *p* < 0.001) and total cholesterol (OR: 1.017, 95% C.I.: 1.007–1.027, *p* = 0.001) were the only factors independently associated with the presence of BMI-SO, but only WC had relatively good discriminative ability (AUC for WC: 0.77, 95% C.I.: 0.71–0.82; AUC for total cholesterol: 0.63, 95% C.I.: 0.57–0.67) (Figure 2). Differences between men and women for the reported variables are presented in Supplementary Table S2, while in multivariate analysis, only WC was independently associated with BMI-SO in both genders.

#### 3.2.2. SO Diagnosed Based on BF% and ALM/W (BF–SO)

**Figure 3:**
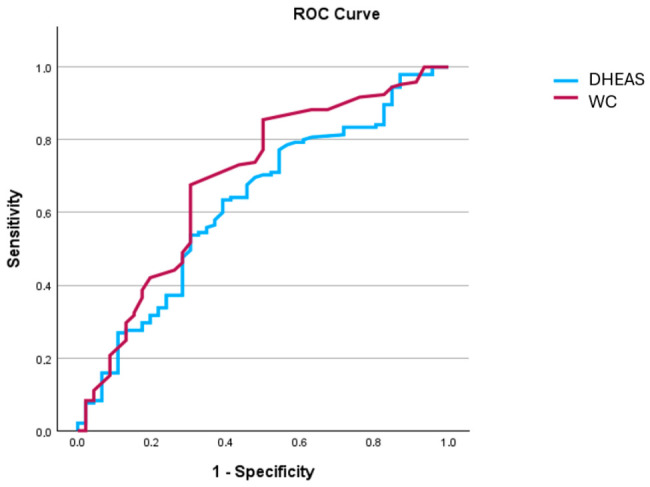
ROC curve for the discriminative ability of waist circumference and dehydroepiandrosterone sulfate for the presence of body fat percentage-based sarcopenic obesity. Abbreviations: ROC: receiver operating characteristic; DHEAS: dehydroepiandrosterone sulfate; WC: waist circumference.

Patients with, compared to those without BF–SO, were older and more frequently women, while they had a higher prevalence of AH. In addition, the former group had higher BMI, WC and BF%, while they had greater levels of fasting Glu, insulin, HOMA-IR index and triglycerides. Regarding hormonal profile, the patients with BF–SO, compared to those without BF–SO, had higher levels of FSH, LH, while they had lower levels of DHEAS. In relation to the severity of liver disease, individuals with BF–SO had worse steatosis based on CAP values (280 vs. 268 dB/m, *p* = 0.013), while they had poorer physical performance based on both SPPB (11 vs. 12, *p* = 0.041) and LFI (3.9 vs. 3.62, *p* = 0.004) (Table 3). In multivariate logistic regression analysis, DHEAS (OR: 0.97, 95% C.I.: 0.96–0.98, *p* = 0.04) and WC (OR: 1.035, 95% C.I.: 1.005–1.065, *p* = 0.022) were the only factors independently associated with BF–SO, although both had relatively low discriminative ability (AUC: 0.65, 95% C.I.: 0.61–0.69 and AUC: 0.68, 95% C.I.: 0.62–0.73, respectively) (Figure 3). After adjusting for Δ4 androstenedione and testosterone, DHEAS (OR: 0.96, 95% C.I.: 0.94–0.98, *p* =0.03) and WC (OR: 1.042, 95% C.I.: 1.007–1.061, *p* =0.031) were again independently associated with BF–SO.

Further analyzing differences by gender, men with BF–SO, compared to those without BF–SO, were older and had higher AH prevalence. In addition, the former group had higher BMI, WC and BF%, while they had greater levels of fasting Glu, insulin, HOMA-IR index, LDL cholesterol and FIB-4. Regarding hormonal profile, men with BF–SO had lower DHEAS levels, compared to those without BF–SO. Multivariate analysis confirmed the findings in the total cohort, since again DHEAS (OR: 0.95, 95% C.I.: 0.91–0.99, *p* = 0.03; after adjustment, OR: 0.94, 95% C.I.: 0.89–0.99, *p* = 0.04) and WC (OR: 1.072, 95% C.I.: 1.015–1.083, *p* = 0.02; after adjustment, OR: 1.066, 95% C.I.: 1.022–1.081, *p* = 0.04) were independently associated with BF–SO, but with low discriminative ability (AUC: 0.62, 95% C.I.: 0.58–0.66 and AUC: 0.67, 95% C.I.: 0.61–0.75, respectively).

Women with BF–SO, compared to those without BF–SO, had higher BMI, WC and BF%, and no significant difference was found regarding the hormonal profile. In relation to the severity of liver disease, women with BF–SO had worse steatosis based on CAP values (277 vs. 255 dB/m, *p* = 0.013), while they had poorer physical performance based on LFI (4.06 vs. 3.69, *p* = 0.018) (Table 4). In multivariate analysis, WC was the only factor independently associated with BF–SO (OR: 1.15, 95% C.I.: 1.092–1.221, *p* = 0.01) with low discriminative ability (AUC: 0.63, 95% C.I.: 0.59–0.67).

#### 3.2.3. SO Diagnosed Based on AIM-SO Score

Patients with SO, compared to those without SO, based on the AIM-SO score, had higher WC, BMI, and BF% and lower values of ALM/W. In addition, the former group had higher LDL cholesterol levels, while they had lower γ-GT. In relation to the severity of liver disease, individuals with AIM-SO had worse steatosis based on CAP values (289 vs. 274 dB/m, *p* = 0.044), while they had poorer physical performance based on LFI (3.95 vs. 3.81, *p* = 0.043) (Supplementary Table S3). In multivariate analysis, WC was the only factor independently associated with AIM-SO (OR: 1.23, 95% C.I.: 1.082–1.345, *p* = 0.02) with relatively good discriminative ability (AUC: 0.78, 95% C.I.: 0.73–0.84).

## 4. Discussion

**Figure 4:**
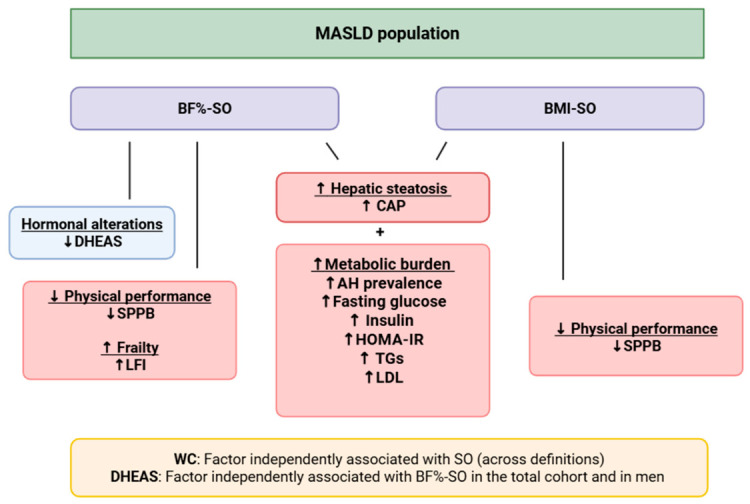
The findings of our study are in line with the proposed hypothetical model linking hormonal alterations with changes in body composition and functional status in patients with MASLD in our study. Lower DHEAS levels may contribute to the development of SO, which appears to be associated with reduced physical performance and increased frailty, increased metabolic burden and hepatic steatosis. DHEAS emerged as a factor independently associated with BF–SO in the total cohort and in men and WC as a factor independently associated with SO across all definitions. Abbreviations: MASLD: metabolic dysfunction-associated steatotic liver disease; BF%: body fat percentage; BMI: body mass index; SO: sarcopenic obesity; CAP: controlled attenuation parameter; AH: arterial hypertension; HOMA-IR: homeostatic model assessment for insulin resistance; TGs: triglycerides; LDL: low density lipoprotein; SPPB: short physical performance battery; LFI: liver frailty index; DHEAS: dehydroepiandrosterone sulfate; WC: waist circumference.

This is the first study, to the best of our knowledge, in which hormonal alterations and physical performance were evaluated in relation to SO in a MASLD population, using different diagnostic criteria for SO. Notably, among the hormones measured, DHEAS emerged as an independent factor associated with BF–SO, while WC was consistently associated with SO across all definitions. In addition, we observed poorer performance in functional tests in individuals with SO, an association not previously examined in patients with MASLD (Figure 4).

Our study attempted to assess the prevalence of SO in MASLD patients, from the Greek population, using different diagnostic criteria, BMI and BF% for assessing obesity, and the recently proposed AIM-SO score. Most available studies in the literature rely on a single SO definition, while many of them focus on Asian populations, making comparisons with Caucasians more challenging [17]. Based on our results, BF–SO showed the highest prevalence, reaching 76.5%, followed by BMI-SO at 51%, further declining to 34.5% when the AIM-SO score was used. These findings confirm the fluctuating prevalence of SO presented in other studies, depending on the diagnostic criteria used and the characteristics of the studied population. Indeed, studies using BF% derived from DEXA generally reported the highest SO prevalence [10,32,33], while BMI-based definitions consistently presented much lower values [34,35]. The different criteria likely capture distinct aspects of body composition, which may explain the variation in SO prevalence. In addition, the relatively higher prevalence of SO that we found −76.5% when defined by BF– may reflect population-specific differences in body composition, as our study, in contrast to others, included exclusively Caucasian individuals with MASLD [10,36]. The variability in prevalence may also be explained by the lack of a universally accepted gold standard for SO, although it seems that BF–criteria appeared to reflect better actual adiposity/body fat and may have higher predictive impact, compared to other definitions [37]. Overall, further research is required to establish the most clinically relevant definition of SO in the context of MASLD.

As expected, SO patients had higher body weight, BMI and BF% compared to those without SO, although these findings were not entirely consistent in the AIM-SO-diagnosed group, suggesting that this criterion may identify a distinct SO phenotype, in which muscle mass and fat distribution are evaluated relative to each other rather than as absolute values. Like previous studies [10,30], we found that MASLD patients with SO more often had metabolic comorbidities, with AH being significantly more prevalent across all definitions. Moreover, regarding glycemic profile, patients with, compared to those without SO (based on BMI or BF%), had higher fasting glycemic levels and insulin resistance, as expressed by the HOMA-IR index, as well as more frequent dyslipidemia as expressed by higher cholesterol and/or triglycerides levels. These results are in accordance with the concept that insulin resistance along with lipotoxicity are key mechanisms in the liver-adipose-muscle axis, playing an essential role in the pathogenesis of both SO and MASLD [38]. This also supports the view that SO may represent a metabolically more vulnerable phenotype within the MASLD.

Regarding liver enzymes (AST, ALT), no significant differences were noted among the groups with or without SO, although individuals with SO across all definitions exhibited significantly greater steatosis as indicated by CAP. These findings confirm previous reports [30] and are consistent with the well-known observation that AST/ALT do not reflect the severity of steatosis. The stronger association between SO and liver steatosis likely reflects the shared pathophysiological background, including central adiposity and insulin resistance, which lead to fat accumulation in the liver. Regarding liver stiffness, it did not appear to be meaningfully worse in the SO groups of our study, which may be explained by the fact that most of our patients had mild liver disease as indicated by low FIB-4 score values and liver stiffness.

Although SO and physical performance have been studied before, especially in older adults [39,40], their relationship had not been investigated in patients with MASLD. In our study, functional performance tests were found to be poorer based on SPPB scores in both BMI-SO and BF–SO groups and on LFI in both BF–SO and AIM-SO-based SO groups. Such observations suggest that functional impairment in SO may be present even in populations with predominantly preserved mobility, as seen in our outpatient cohort. Reduced physical performance is clinically important as it is linked to a higher risk of frailty, falls and worse overall outcomes [41]. Therefore, detecting lower SPPB and LFI scores may represent an early manifestation of muscle dysfunction, while these simple functional tests might serve as valuable indicators of SO in MASLD patients who otherwise appear physically well.

Hormonal factors, such as testosterone, appear to be implicated in the pathogenesis of SO, influencing muscle mass and metabolic health [16]. In our study, we evaluated for the first time not only total and free testosterone, but also DHEAS and Δ4 in the context of SO and found that lower DHEAS levels were independently associated with the presence of BF–SO. However, DHEAS had modest AUC values in our analysis. BF–SO may better reflect true adiposity compared to other criteria and may therefore be more sensitive to sex-related differences in body composition and endocrine function. In our sex-stratified analyses, we confirmed these findings in men, supporting a sex-specific influence in BF–SO, although these analyses are exploratory given the limited sample size. DHEAS represents a key precursor that is converted to Δ4 and ultimately to testosterone [42,43], and has been shown to influence adipose tissue metabolism, including lipolysis, insulin sensitivity and adipocyte differentiation, while also affecting muscle metabolism and inflammatory pathways [44,45]. Therefore, alterations in this hormone may be associated with the presence of SO, particularly in BF–based definitions that directly reflect adiposity and could serve as a potential indicator of SO in individuals with MASLD. Our findings are also in line with the proposed conceptual model linking hormonal alterations, body composition, physical performance, and frailty in MASLD (Figure 4). However, since DHEAS is influenced by age, sex, liver function, and overall health, this association should be viewed as observational. Moreover, the association we found was modest and the discriminative ability was limited, indicating limited value as a standalone clinical marker. These results should be interpreted with caution in the broader endocrine context. Additionally, even though we did not find associations with other hormones such as testosterone, these pathways may still play a role, warranting further investigation in both men and women.

Moreover, as seen in the multivariate analyses, WC emerged as the factor more consistently associated with SO across all definitions. A similar pattern was shown in the sex-specific sub-analyses, with WC acting as an independent factor in both men and women. These findings highlight the central role of abdominal fat accumulation in the relationship between muscle mass, metabolism, and liver disease and in all forms of SO. From a pathophysiological perspective, the hypertrophic and dysfunctional adipose tissue represents a pro-inflammatory state and is responsible for cytokine secretion, such as tumor necrosis factor-α (TNF-α) and interleukin-6 (IL-6) [46]. Such molecules not only trigger inflammatory pathways and hepatic fibrogenesis but also promote muscle catabolism, further exacerbate insulin resistance and interfere with the physiological function of the liver-adipose-muscle axis, ultimately contributing to SO and MASLD development [16]. The fact that WC primarily reflects visceral adiposity, which is actively involved in insulin resistance, inflammation and metabolic regulation, explains its consistent association across all definitions of SO. Therefore, measuring and assessing WC in MASLD patients may represent an easily obtainable clinical parameter associated with SO in MASLD patients.

We acknowledge certain limitations of our study. First, this was a single-center study, and the sample size was relatively small, while we mainly showed associations among SO and comorbidities and did not thoroughly explore causality in these relationships. Most of the participants had preserved mobility and had relatively mild liver disease with few cases of advanced fibrosis, thus perhaps over- or under-estimating the true prevalence and metabolic impact of SO in more advanced stages of MASLD. Accordingly, the low burden of frailty and mainly preserved mobility may have introduced a ceiling effect, limiting the sensitivity of frailty assessment. However, a considerable proportion of patients were classified as pre-frail, suggesting that early functional decline may already be present and underlining the importance of early identification and prevention. Therefore, our results may not be fully generalizable to individuals with more advanced fibrosis or greater functional impairment. Another limitation was the lack of a universally accepted gold standard for SO, which contributed to the variability in its prevalence across definitions. Direct measurement of skeletal muscle volume, such as the skeletal muscle index (SMI), was not available in our cohort and could perhaps provide complementary information in future studies. Nevertheless, CT has the limitation of radiation exposure, and this was a main reason to choose DEXA for ALMI assessment, while, based on current guidelines, it is generally a less expensive technique as well as a very reliable and alternative method for muscle mass evaluation, compared to a CT scan [47]. The exploratory nature of the statistical analysis should also be acknowledged. Stepwise logistic regression was used as a variable-reduction approach, with candidate variables selected according to clinical relevance, biological plausibility, univariate associations, and avoidance of collinearity. Nevertheless, this approach may be associated with model instability and overfitting. Moreover, multiple comparisons were performed across different SO definitions and sex-specific subgroup analyses, without formal adjustment for multiple testing. Therefore, *p*-values should be interpreted with caution, and the findings should be considered hypothesis-generating and validated in larger cohorts. Our study also has several strengths, including the use of DEXA for assessing body composition, CAP to evaluate steatosis and liver stiffness to quantify fibrosis, while three different definitions were applied to define SO. To the best of our knowledge, this is the first study evaluating the physical performance/frailty among MASLD patients with and without SO, while also examining related hormonal alterations in this population.

## 5. Conclusions

In conclusion, SO prevalence among Caucasian patients with MASLD appears to be high, particularly when defined by BF% and is associated with greater hepatic steatosis and metabolic comorbidities, as well as poorer physical performance. Low DHEAS levels were associated with BF–SO in the total population, as well as in men, but not in women, although the sex-stratified analyses were exploratory in nature. Nevertheless, the relatively low discriminative ability of DHEAS suggests that it may be better regarded as a potential and supportive biomarker that requires further validation rather than a standalone risk indicator, while WC was repeatedly found to be linked to SO regardless of definition. Our findings contribute to the understanding of SO in MASLD, which may help improve its early recognition, ultimately enabling more accurate clinical assessment and management of these patients. At the same time, the need for further research is highlighted to validate these findings and clarify their clinical relevance, as well as the impact of different SO definitions on the observed associations.

## Figures and Tables

**Table 1:** Main demographic, clinical and laboratory characteristics of the population.

	Total Participants (*n* = 200)
Age, years	55.86 (±13.69)
Sex, males, *n* (%)	91 (45.5)
Comorbidities	
T2D, *n* (%)	55 (28.1)
AH, *n* (%)	72 (36.9)
Dyslipidemia, *n* (%)	98 (49.5)
Obesity (BMI), (%)	113 (56.5)
Obesity (BF%), (%)	185 (92.5)
Low ALM/W, (%)	160 (80)
WC, cm	105.07 (±13.54)
ΒΜΙ, kg/m^2^	31.70 (±5.68)
BF%	43.41 (±7.06)
ALM/W, %	23.84 (±4.24)
AIMSO score	0.40 (±0.13)
BMI-SO, *n* (%)	102 (51)
BF–SO, *n* (%)	153 (76.5)
SO based on AIM-SO score, *n* (%)	69 (34.5)
HbA1c (%)	5.91 (±0.80)
Glu, mg/dL	93.18 (±23.59)
Insulin, μU/mL	16.10 (±12.20)
HOMA-IR	3.92 (±3.73)
Total cholesterol, mg/dL	190.53 (±40.85)
HDL cholesterol, mg/dL	52.12 (±13.07)
LDL cholesterol, mg/dL	111.71 (±36.49)
Triglycerides, mg/dL	127.30 (±58.29)
AST, U/L	30.00 (±21.09)
ALT, U/L	39.63 (±36.83)
γ-GT, U/L	61.13 (±74.03)
25(OH)VitD, ng/mL	24.98 (±10)
Ferritin, ng/mL	158.72 (±177.54)
**Hormonal status**	
FSH, mIU/mL	46.30 (±51.56)
LH, mIU/mL	15.56 (±13.97)
DHEAS, μg/dL	147.58 (±130.02)
SHBG, nmol/L	44.87 (±21.52)
Testo, ng/mL	6.78 (±52.93)
Free Testo, ng/mL	0.21 (±1.97)
Δ4, ng/mL	0.91 (±0.8)
**Severity of liver disease**	
FIB-4	1.19 (±0.90)
CAP, dB/m	287.79 (±41.37)
Liver stiffness, kPa	6.95 (±5.07)
**Functional performance tests**	
SPPB	10.98 (±1.21)
LFI	3.82 (±0.58)

Continuous variables are expressed as mean (SD) and categorical variables as the number of individuals *n* (percentage of the sample, %). Abbreviations: T2D: type 2 diabetes; AH: arterial hypertension; BMI: body mass index; BF%: body fat percentage; ALM/W: appendicular lean mass-to-weight ratio; SO: sarcopenic obesity; WC: waist circumference; HbA1c: hemoglobin A1c; Glu: fasting glucose; HOMA-IR: homeostatic model assessment of insulin; HDL: high density lipoprotein; LDL: low-density lipoprotein; AST: aspartate aminotransferase; ALT: alanine aminotransferase; γ-GT: gamma-glutamyl transferase; 25(OH)VitD: 25-hydroxy-vitamin D; FSH: follicle-stimulating hormone; LH: luteinizing hormone; DHEAS: dehydroepiandrosterone sulfate; SHBG: sex hormone-binding globulin; Testo: testosterone; Δ4: Δ4 androstenedione; FIB-4: fibrosis-4 index; CAP: controlled attenuation parameter; SPPB: short physical performance battery; LFI: liver frailty index.

**Table 2:** Comparison of patients with and without BMI-SO (univariate and multivariate analysis).

	Without BMI-SO *n* = 98 (49%)	With BMI-SO *n* = 102 (51%)	*p*-Value	OR	95% C.I.	*p* Value
	Univariate Analysis			Multivariate Analysis		
Age, years	55 (46.25–64.75)	59.50 (50.25–66.75)	0.740			
Sex, male, *n* (%)	48 (48.98)	43 (42.16)	0.333			
Cormobidities						
T2D, *n* (%)	23 (24.2)	32 (31.6)	0.245			
AH, *n* (%)	27 (28.7)	45 (44.5)	**0.022**			
Dyslipidemia, *n* (%)	53 (54.6)	45 (44.5)	0.156			
WC, cm	97 (90.25–103)	110 (103–119)	**<0.001**	**1.085**	**1.047–1.125**	**<0.001**
BMI, kg/m^2^	27.85 (26.22–29.17)	34.05 (33–37.35)	**<0.001**			
BF%	38.45 (34.62–45.07)	48.95 (42.1–50.5)	**<0.001**			
ALM/W, (%)	25.42 (22.3–28.81)	21.6 (20.06–25.14)	**<0.001**			
HbA1c (%)	5.65 (5.4–6.1)	5.7 (5.4–6)	0.159			
Glu, mg/dL	86 (78.25–98.75)	88 (81.25–102.5)	**0.030**			
Insulin, μU/mL	11.3 (7.3–18.32)	14.6 (11.52–20.65)	**<0.001**			
HOMA-IR	2.57 (1.31–4.13)	3.25 (2.27–5.09)	**<0.001**			
Total cholesterol, mg/dL	178.5 (153.25–206.5)	194 (166.25–226)	**0.043**	**1.017**	**1.007–1.027**	**0.001**
HDL cholesterol, mg/dL	52.97 (±13.15)	51.27 (±13.00)	0.380			
LDL cholesterol, mg/dL	101.5 (79–134.75)	114.5 (87.75–144.5)	**0.040**			
Triglycerides, mg/dL	107 (79–138)	126.5 (93–161.25)	**0.044**			
AST, U/L	23 (20–29)	21 (18–31.75)	0.958			
ALT, U/L	28 (19–43.75)	24.5 (18.25–38.75)	0.375			
γ-GT, U/L	31 (21–78.75)	25 (17.25–67)	0.794			
25(OH)VitD, ng/mL	24.5 (19.05–31.8)	24.5 (19.2–30.7)	0.876			
Ferritin, ng/mL	111 (62.2–202)	104 (62.8–160)	0.511			
**Hormonal status**						
FSH, mIU/mL	11.4 (6.87–66.25)	41.1 (8.28–85.5)	0.227			
LH, mIU/mL	6.78 (3.18–22.3)	14.3 (4.31–27.5)	0.060			
DHEAS, μg/dL	121.35 (63.5–214)	105 (55.82–180.5)	0.644			
SHBG, nmol/L	46.69 (±20.09)	43.07 (±22.82)	0.261			
Testo, ng/mL	2.96 (0.22–4.61)	0.46 (0.13–4.09)	0.055			
Free Testo, ng/mL	0.021 (0.003–0.08)	0.009 (0.002–0.07)	0.694			
Δ4, ng/mL	0.75 (0.38–1.07)	0.54 (0.3–0.92)	0.531			
**Severity of liver disease**						
FIB-4	0.98 (0.71–1.43)	1.04 (0.68–1.40)	0.731			
CAP, dB/m	270 (249.5–283)	292.5 (262.25–337.5)	**<0.001**			
Liver stiffness, kPa	5.42 (4.81–6.71)	5.63 (4.7–6.45)	0.347			
**Functional performance tests**						
SPPB	12 (11–12)	11 (10–12)	**0.002**			
LFI	3.83 (3.39–4.18)	3.84 (3.47–4.29)	0.284			

Continuous variables expressed as mean (SD) or median with interquartile range (IQR: 25th–75th percentile) and categorical variables as number of individuals *n* (percentage of the sample, %). Bold numerical values indicate statistical significance (*p* < 0.05). Abbreviations: T2D: type 2 diabetes; AH: arterial hypertension; BMI: body mass index; BF%: body fat percentage; ALM/W: appendicular lean mass-to-weight ratio; SO: sarcopenic obesity; WC: waist circumference; HbA1c: hemoglobin A1c; Glu: fasting glucose; HOMA-IR: homeostatic model assessment of insulin; HDL: high density lipoprotein; LDL: low-density lipoprotein; AST: aspartate aminotransferase; ALT: alanine aminotransferase; γ-GT: gamma-glutamyl transferase; 25(OH)VitD: 25-hydroxy-vitamin D; FSH: follicle-stimulating hormone; LH: luteinizing hormone; DHEAS: dehydroepiandrosterone sulfate; SHBG: sex hormone-binding globulin; Testo: testosterone; Δ4: Δ4 androstenedione; FIB-4: fibrosis-4 index; CAP: controlled attenuation parameter; SPPB: short physical performance battery; LFI: liver frailty index.

**Table 3:** Comparison of patients with and without BF–SO (univariate and multivariate analysis).

	Without BF–SO *n* = 47 (23.5%)	With BF–SO *n* = 153 (76.5%)	*p*-Value	OR	95% C.I.	*p* Value
	Univariate Analysis			Multivariate Analysis		
Age, years	47 (37–56)	59 (52–67)	**0.003**			
Sex, male, *n* (%)	29 (61.7)	62 (40.5)	**0.011**			
Cormobidities						
T2D, *n* (%)	10 (21.7)	45 (30)	0.275			
AH, *n* (%)	11 (23.9)	61 (40.9)	**0.036**			
Dyslipidemia, *n* (%)	21 (44.7)	77 (51)	0.450			
WC, cm	93 (90–107)	104 (97.5–113)	**<0.001**	**1.035**	**1.005–1.065**	**0.022**
BMI, kg/m^2^	26.6 (24.8–28.7)	32.4 (28.95–35.05)	**<0.001**			
BF%	34.8 (32.9–38.9)	45.8 (40.2–50.1)	**<0.001**			
ALM/W, (%)	28.98 (27.73–30.11)	22.4 (20.5–25.62)	**<0.001**			
HbA1c (%)	5.4 (5.3–6.1)	5.7 (5.4–6.05)	0.168			
Glu, mg/dL	82 (72–92)	88 (81–102.5)	**0.005**			
Insulin, μU/mL	9.68 (6.91–18)	13.7 (9.62–19)	**0.001**			
HOMA-IR	1.7 (1.21–3.84)	3.17 (1.83–4.67)	**<0.001**			
Total cholesterol, mg/dL	181 (148–226)	191 (160.5–223.5)	0.216			
HDL cholesterol, mg/dL	47 (42.3–62.1)	51.5 (43.5–62.25)	0.558			
LDL cholesterol, mg/dL	103 (78–145)	110 (84.5–142)	0.507			
Triglycerides, mg/dL	104 (71–136)	121 (92.5–148)	**0.040**			
AST, U/L	23 (20–33)	22 (19–30)	0.757			
ALT, U/L	31 (21–47)	25 (18–40)	0.961			
γ-GT, U/L	32 (21–79)	25 (18–68.5)	0.622			
25(OH)VitD, ng/mL	22 (16.85–29.05)	25 (19.9–31.6)	0.306			
Ferritin, ng/mL	114 (43.75–213.5)	108 (65.3–162)	0.915			
**Hormonal status**						
FSH, mIU/mL	8.63 (5.72–14.9)	45.4 (8.45–85.5)	**<0.001**			
LH, mIU/mL	4.78 (2.83–10.8)	16.9 (4.34–27.4)	**<0.001**			
DHEAS, μg/dL	166 (101–299)	103 (54.2–179.5)	**0.028**	**0.97**	**0.96–0.98**	**0.04**
SHBG, nmol/L	41.3 (25.9–52.7)	42.9 (30.45–58)	0.482			
Testo, ng/mL	3.74 (0.25–4.53)	0.48 (0.18–4.32)	0.138			
Free Testo, ng/mL	0.05 (0.003–0.1)	0.006 (0.002–0.07)	0.085			
Δ4, ng/mL	0.8 (0.35–1.21)	0.56 (0.3–0.96)	0.235			
**Severity of liver disease**						
FIB-4	0.89 (0.61–1.13)	1.06 (0.77–1.47)	0.126			
CAP, dB/m	268 (246–284)	280 (256–322.5)	**0.013**			
Liver stiffness, kPa	5.61 (4.93–7.1)	5.45 (4.67–4.35)	1.000			
**Functional performance tests**						
SPPB	12 (11–12)	11 (10–12)	**0.041**			
LFI	3.62 (3.3–3.97)	3.9 (3.5–4.28)	**0.004**			

Continuous variables expressed as mean (SD) or median with interquartile range (IQR: 25th–75th percentile) and categorical variables as number of individuals *n* (percentage of the sample, %). Bold numerical values indicate statistical significance (*p* < 0.05). Abbreviations: T2D: type 2 diabetes; AH: arterial hypertension; BMI: body mass index; BF%: body fat percentage; ALM/W: appendicular lean mass-to-weight ratio; SO: sarcopenic obesity; WC: waist circumference; HbA1c: hemoglobin A1c; Glu: fasting glucose; HOMA-IR: homeostatic model assessment of insulin; HDL: high density lipoprotein; LDL: low-density lipoprotein; AST: aspartate aminotransferase; ALT: alanine aminotransferase; γ-GT: gamma-glutamyl transferase; 25(OH)VitD: 25-hydroxy-vitamin D; FSH: follicle-stimulating hormone; LH: luteinizing hormone; DHEAS: dehydroepiandrosterone sulfate; SHBG: sex hormone-binding globulin; Testo: testosterone; Δ4: Δ4 androstenedione; FIB-4: fibrosis-4 index; CAP: controlled attenuation parameter; SPPB: short physical performance battery; LFI: liver frailty index.

**Table 4:** Comparison of patients with and without BF–SO, by gender (univariate and multivariate analysis).

	Men				Women			
	Patients Without BF–SO (*n* = 29)	Patients with BF–SO (*n* = 62)	*p*-Value		Patients Without BF–SO (*n* = 18)	Patients with BF–SO (*n* = 91)	*p*-Value	
	Univariate Analysis			Multivariate Analysis (OR, 95% C.I., *p* Value)	Univariate Analysis			Multivariate Analysis (OR, 95% C.I., *p* Value)
Age, years	47.93 (±13.81)	56.23 (±15.71)	**0.017**		54.94 (±14.15)	58.31 (±11.1)	0.265	
Cormobidities								
T2D, *n* (%)	7 (24.1)	17 (27.4)	0.744		3 (16.6)	28 (30.7)	0.216	
AH, *n* (%)	5 (17.2)	28 (45.2)	**0.009**		6 (33.3)	33 (36.2)	0.763	
Dyslipidemia, *n* (%)	14 (48.3)	28 (45.2)	0.833		7 (38.8)	49 (53.8)	0.228	
WC, cm	99.5 (90.75–107.25)	108 (100–119)	**<0.001**	**1.072, 1.015–1.083, *p* = 0.02**	91.12 (±9.25)	104.61 (±10.24)	<0.001	**1.15, 1.092–1.221, *p* = 0.01**
BMI, kg/m^2^	27.21 (±3.81)	32.77 (±5.02)	**<0.001**		26.3 (24.8–30.45)	33.2 (28.7–35.8)	**<0.001**	
BF%	33.65 (32.05–35)	37.75 (36.67–42.5)	**<0.001**		40.91 (±3.91)	49.19 (±3.8)	**<0.001**	
ALM/W, (%)	29.28 (28.85–30.31)	25.83 (23.79–27.13)	**<0.001**		27.42 (24.45–30.27)	21.14 (19.57–22.19)	**<0.001**	
HbA1c (%)	5.4 (5.3–6.17)	5.6 (5.4–6.02)	0.296		5.8 (5.45–6.25)	5.8 (5.4–6.1)	0.509	
Glu, mg/dL	81 (66.25–96.5)	88 (82.25–100.25)	**0.021**		89.24 (±19.95)	97.14 (±24.3)	0.211	
Insulin, μU/mL	9.72 (7.18–14.15)	14.5 (10.27–24.02)	**0.005**		11.11 (±7.53)	16.5 (±12.49)	0.099	
HOMA-IR	2.03 (1.15–3.12)	3.2 (1.94–5.49)	**0.002**		2.6 (±2.14)	4.22 (±4.19)	0.148	
Total cholesterol, mg/dL	176.61 (±35.17)	191.29 (±34.06)	0.073		174 (153–235)	180 (153–203)	0.912	
HDL cholesterol, mg/dL	47.51 (±10.32)	48.98 (±12.06)	0.593		56.98 (±13.51)	54.56 (±13.64)	0.503	
LDL cholesterol, mg/dL	104.78 (±30.38)	121.06 (±30.86)	**0.031**		95 (82–147.5)	100 (79–119)	0.484	
Triglycerides, mg/dL	115 (68.75–129.25)	103.5 (90.75–159)	0.490		91 (64–160.5)	115 (91–137)	0.095	
AST, U/L	22 (20–27.75)	23 (19–38.5)	0.306		22 (19.5–31.5)	22 (16–28)	0.805	
ALT, U/L	30 (21–50.5)	36 (19.75–57)	0.860		21 (15.5–32)	24 (16–31)	0.414	
γ-GT, U/L	32.5 (17.25–63.25)	37 (24.75–94)	0.490		32 (16.5–70.5)	24 (16–48)	0.449	
25(OH)VitD, ng/mL	24.20 (±10.05)	23.91 (±9.01)	0.898		21.3 (13.15–28.2)	25.9 (19.2–33.6)	0.173	
Ferritin, ng/mL	145.5 (46.9–280)	131 (79.17–212.5)	0.913		79.3 (33.75–152.5	86.9 (49.5–145)	0.335	
**Hormonal status**								
FSH, mIU/mL	6.57 (3.73–6.57)	7.12 (4.96–16.92)	0.063		59.6 (10.5–65.72)	79.4 (46.55–95.62)	0.140	
LH, mIU/mL	3.35 (2.58–6.14)	4.17 (3.14–6.81)	0.055		14.3 (6.11–25.35)	24.9 (16.92–32.27)	0.060	
DHEAS, μg/dL	217.5 (152.25–331.30)	117.5 (76.22–137.5)	**0.028**	**0.95, 0.91–0.99, *p* = 0.03**	103 (45.67–171.5)	100 (54.1–151.25)	0.593	
SHBG, nmol/L	37.86 (±17.28)	39.33 (±18.86)	0.739		50.73 (±22.6)	49.42 (±22.93)	0.834	
Testosterone, ng/mL	4.43 (3.8–6.58)	4 (2.64–4.94)	0.062		0.18 (0.13–0.27)	0.19 (0.13–0.41)	0.068	
Free testo, ng/mL	0.05 (0.004–0.1)	0.02 (0.002–0.069)	0.212		0.007 (0.003–0.11)	0.006 (0.002 -0.07)	0.278	
Δ4, ng/mL	0.83 (0.51–1.28)	0.61 (0.48–1.06)	0.683		0.9 (0.33–1.99)	0.58 (0.3–0.99)	0.330	
**Severity of liver disease**								
FIB-4	0.89 (0.61–1.14)	1.14 (0.81–1.57)	**0.004**		1.04 (0.89–1.43)	1.02 (0.8–1.5)	0.188	
CAP, dB/m	279.46 (±34.8)	297.8 (±45.54)	0.066		255 (233.5–269)	277 (254–298)	**0.013**	
Liver stiffness, kPa	6.02 (5.27–7.44)	5.75 (4.98–6.66)	0.827		5 (4.55 -6.31)	5.3 (4.55–6.3)	0.603	
**Functional performance tests**								
SPPB	12 (11–12)	12 (11–12)	0.211		12 (10.5–12)	11 (10–12)	0.286	
LFI	3.48 (±0.69)	3.69 (±0.51)	0.171		3.69 (±0.51)	4.06 (±0.51)	**0.018**	

Continuous variables expressed as mean (SD) or median with interquartile range (IQR: 25th–75th percentile) and categorical variables as number of individuals *n* (percentage of the sample, %). Bold numerical values indicate statistical significance (*p* <0.05). Abbreviations: T2D: type 2 diabetes; AH: arterial hypertension; BMI: body mass index; BF%: body fat percentage; ALM/W: appendicular lean mass-to-weight ratio; SO: sarcopenic obesity; WC: waist circumference; HbA1c: hemoglobin A1c; Glu: fasting glucose; HOMA-IR: homeostatic model assessment of insulin; HDL: high density lipoprotein; LDL: low-density lipoprotein; AST: aspartate aminotransferase; ALT: alanine aminotransferase; γ-GT: gamma-glutamyl transferase; 25(OH)VitD: 25-hydroxy-vitamin D; FSH: follicle-stimulating hormone; LH: luteinizing hormone; DHEAS: dehydroepiandrosterone sulfate; SHBG: sex hormone-binding globulin; Testo: testosterone; Δ4: Δ4 androstenedione; FIB-4: fibrosis-4 index; CAP: controlled attenuation parameter; SPPB: short physical performance battery; LFI: liver frailty index.

## Data Availability

The original contributions presented in this study are included in the article/Supplementary Materials. Further inquiries can be directed to the corresponding author(s).

## Supplementary Materials

The following supporting information can be downloaded at: https://www.mdpi.com/article/10.3390/medsci14020257/s1, Supplementary Table S1: Medication used for AH, dyslipidemia, and T2D in our population, Supplementary Table S2: Comparison of patients with and without BMI-SO, by gender (univariate and multivariate analysis), Supplementary Table S3: Comparison of patients with and without SO - based on AIMSO score.

## References

[1] Rinella M.E., Lazarus J.V., Ratziu V., Francque S.M., Sanyal A.J., Kanwal F., Romero D., Abdelmalek M.F., Anstee Q.M., Arab J.P. (2023). A multisociety Delphi consensus statement on new fatty liver disease nomenclature. Hepatology.

[2] Tacke F., Horn P., Wai-Sun Wong V., Ratziu V., Bugianesi E., Francque S., Zelber-Sagi S., Valenti L., Roden M., Schick F. (2024). EASL–EASD–EASO Clinical Practice Guidelines on the management of metabolic dysfunction-associated steatotic liver disease (MASLD). J. Hepatol..

[3] Le M.H., Yeo Y.H., Zou B., Barnet S., Henry L., Cheung R., Nguyen M.H. (2022). Forecasted 2040 global prevalence of nonalcoholic fatty liver disease using hierarchical bayesian approach. Clin. Mol. Hepatol..

[4] Quek J., Chan K.E., Wong Z.Y., Tan C., Tan B., Lim W.H., Tan D.J.H., Tang A.S.P., Tay P., Xiao J. (2023). Global prevalence of non-alcoholic fatty liver disease and non-alcoholic steatohepatitis in the overweight and obese population: A systematic review and meta-analysis. Lancet Gastroenterol. Hepatol..

[5] Cruz-Jentoft A.J., Bahat G., Bauer J., Boirie Y., Bruyère O., Cederholm T., Cooper C., Landi F., Rolland Y., Sayer A.A. (2019). Sarcopenia: Revised European consensus on definition and diagnosis. Age Ageing.

[6] Yuan S., Larsson S.C. (2023). Epidemiology of sarcopenia: Prevalence, risk factors, and consequences. Metabolism.

[7] Li X., He J., Sun Q. (2024). The prevalence and effects of sarcopenia in patients with metabolic dysfunction-associated steatotic liver disease (MASLD): A systematic review and meta-analysis. Clin. Nutr..

[8] Wong R., Yuan L.Y. (2024). Sarcopenia and metabolic dysfunction associated steatotic liver disease: Time to address both. World J. Hepatol..

[9] Gao Q., Mei F., Shang Y., Hu K., Chen F., Zhao L., Ma B. (2021). Global prevalence of sarcopenic obesity in older adults: A systematic review and meta-analysis. Clin. Nutr..

[10] Chien S.C., Chiu H.C., Chiu Y.C., Wang R.H., Dillera K.P.O., Lee K.T., Tsai H.-W., Tsai Y.-S., Ou H.-Y., Cheng P.-N. (2025). Clinical Relevancies of Sarcopenic Obesity in Patients with Metabolic Dysfunction-Associated Fatty Liver Disease (MASLD). Dig. Dis. Sci..

[11] Donini L.M., Busetto L., Bischoff S.C., Cederholm T., Ballesteros-Pomar M.D., Batsis J.A., Bauer J.M., Boirie Y., Cruz-Jentoft A.J., Dicker D. (2022). Definition and Diagnostic Criteria for Sarcopenic Obesity: ESPEN and EASO Consensus Statement. Obes. Facts.

[12] Tian S., Xu Y. (2016). Association of sarcopenic obesity with the risk of all-cause mortality: A meta-analysis of prospective cohort studies. Geriatr. Gerontol. Int..

[13] Luo Y., Xin C., Liu Y., Xu Y., Liu G., Han B. (2025). Association between sarcopenic obesity and cardiovascular diseases: The role of systemic inflammation indices. Front. Med..

[14] Polyzos S.A., Margioris A.N. (2018). Sarcopenic obesity. Hormones.

[15] Koliaki C., Liatis S., Dalamaga M., Kokkinos A. (2019). Sarcopenic Obesity: Epidemiologic Evidence, Pathophysiology, and Therapeutic Perspectives. Curr. Obes. Rep..

[16] Batsis J.A., Villareal D.T. (2018). Sarcopenic obesity in older adults: Aetiology, epidemiology and treatment strategies. Nat. Rev. Endocrinol..

[17] Wang P., Liu X., Du X., Qiu L., Liu Y., Xu S., Zhang Y., Zhang J. (2025). Prevalence and risk of metabolic dysfunction-associated steatotic liver disease in patients with sarcopenic obesity: A systematic review and meta-analysis. Nutr. Metab..

[18] Song W., Yoo S.H., Jang J., Baik S.J., Lee B.K., Lee H.W., Park J.S. (2023). Association between Sarcopenic Obesity Status and Nonalcoholic Fatty Liver Disease and Fibrosis. Gut Liver.

[19] Wijarnpreecha K., Aby E.S., Ahmed A., Kim D. (2021). Association between Sarcopenic Obesity and Nonalcoholic Fatty Liver Disease and Fibrosis detected by Fibroscan. J. Gastrointestin Liver Dis..

[20] Chun H.S., Lee M., Lee H.A., Lee S., Kim S., Jung Y.J., Lee C., Kim H., Lee H.A., Kim H.Y. (2023). Risk Stratification for Sarcopenic Obesity in Subjects With Nonalcoholic Fatty Liver Disease. Clin. Gastroenterol. Hepatol..

[21] Veronese N., Ragusa F.S., Pegreffi F., Dominguez L.J., Barbagallo M., Zanetti M., Cereda E. (2024). Sarcopenic obesity and health outcomes: An umbrella review of systematic reviews with meta-analysis. J. Cachexia Sarcopenia Muscle.

[22] Matthews D.R., Hosker J.P., Rudenski A.S., Naylor B.A., Treacher D.F., Turner R.C. (1985). Homeostasis model assessment: Insulin resistance and beta-cell function from fasting plasma glucose and insulin concentrations in man. Diabetologia.

[23] Vermeulen A., Verdonck L., Kaufman J.M. (1999). A critical evaluation of simple methods for the estimation of free testosterone in serum. J. Clin. Endocrinol. Metab..

[24] Cuomo G., Iandoli C., Galiero R., Caturano A., Di Vico C., Perretta D., Adamo P.V., Ferrara R., Rinaldi L., Romano C. (2023). Liver Involvement in Patients with Systemic Sclerosis: Role of Transient Elastography in the Assessment of Hepatic Fibrosis and Steatosis. Diagnostics.

[25] Ferraioli G., Parekh P., Levitov A.B., Filice C. (2014). Shear wave elastography for evaluation of liver fibrosis. J. Ultrasound Med..

[26] Sterling R.K., Lissen E., Clumeck N., Sola R., Correa M.C., Montaner J., Sulkowski M.S., Torriani F.J., Dieterich D.T., Thomas D.L. (2006). Development of a simple noninvasive index to predict significant fibrosis in patients with HIV/HCV coinfection. Hepatology.

[27] Guralnik J.M., Ferrucci L., Pieper C.F., Leveille S.G., Markides K.S., Ostir G.V., Studenski S., Berkman L.F., Wallace R.B. (2000). Lower extremity function and subsequent disability: Consistency across studies, predictive models, and value of gait speed alone compared with the short physical performance battery. J. Gerontol. A Biol. Sci. Med. Sci..

[28] Wang C.W., Lebsack A., Chau S., Lai J.C. (2019). The Range and Reproducibility of the Liver Frailty Index. Liver Transpl..

[29] (2000). Obesity: Preventing and Managing the Global Epidemic.

[30] Zambon Azevedo V., Bel Lassen P., Aron-Wisnewsky J., Genser L., Charlotte F., Bedossa P., Ponnaiah M., Pais R., Clément K., Oppert J.-M. (2024). Metabolic and hepatic phenotypes in sarcopenic obesity and impact of bariatric surgery. Clin. Nutr..

[31] Hanley J.A., McNeil B.J. (1982). The meaning and use of the area under a receiver operating characteristic (ROC) curve. Radiology.

[32] Ishimoto R., Mutsuzaki H., Shimizu Y., Kishimoto H., Takeuchi R., Hada Y. (2023). Prevalence of Sarcopenic Obesity and Factors Influencing Body Composition in Persons with Spinal Cord Injury in Japan. Nutrients.

[33] Diago-Galmés A., Guillamon-Escudero C., Tenías-Burillo J.M., Soriano J.M., Fernández-Garrido J. (2023). Sarcopenic Obesity in Community-Dwelling Spanish Adults Older than 65 Years. Nutrients.

[34] Khor E.Q., Lim J.P., Tay L., Yeo A., Yew S., Ding Y.Y., Lim W.S. (2020). Obesity Definitions in Sarcopenic Obesity: Differences in Prevalence, Agreement and Association with Muscle Function. J. Frailty Aging.

[35] Hernández-Martínez P., Olmos J.M., Llorca J., Hernández J.L., González-Macías J. (2022). Sarcopenic osteoporosis, sarcopenic obesity, and sarcopenic osteoporotic obesity in the Camargo cohort (Cantabria, Spain). Arch. Osteoporos..

[36] Elsabaawy M., Ragab A., Abd-Elrazek A., Atef M., Naguib M. (2025). Sarcopenic visceral obesity in patients with metabolic dysfunction-associated steatotic liver disease (MASLD). Clin. Exp. Med..

[37] Mainous A.G., Yin L., Wu V., Sharma P., Jenkins B.M., Saguil A.A., Nelson D.S., Orlando F.A. (2025). Body Mass Index vs Body Fat Percentage as a Predictor of Mortality in Adults Aged 20–49 Years. Ann. Fam. Med..

[38] Polyzos S.A., Vachliotis I.D., Mantzoros C.S. (2023). Sarcopenia, sarcopenic obesity and nonalcoholic fatty liver disease. Metabolism.

[39] Saito H., Matsue Y., Kamiya K., Kagiyama N., Maeda D., Endo Y., Ueno H., Yoshioka K., Mizukami A., Saito K. (2022). Sarcopenic obesity is associated with impaired physical function and mortality in older patients with heart failure: Insight from FRAGILE-HF. BMC Geriatr..

[40] Kong H.H., Won C.W., Kim W. (2020). Effect of sarcopenic obesity on deterioration of physical function in the elderly. Arch. Gerontol. Geriatr..

[41] Ghiotto L., Muollo V., Tatangelo T., Schena F., Rossi A.P. (2022). Exercise and physical performance in older adults with sarcopenic obesity: A systematic review. Front. Endocrinol..

[42] Leowattana W. (2004). DHEAS as a new diagnostic tool. Clin. Chim. Acta.

[43] Adamantou M., Pergantina E., Kamiliou A., Rachiotis N., Lekakis V., Chrysavgis L., Papatheodoridis G., Angelousi A., Cholongitas E. (2025). Association of Hormonal Status With Sarcopenia, Frailty and Outcome in Patients with Decompensated Cirrhosis. Liver Int..

[44] Karbowska J., Kochan Z. (2013). Effects of DHEA on metabolic and endocrine functions of adipose tissue. Horm. Mol. Biol. Clin. Investig..

[45] Lin H.Y., Chen J.H., Chen K.H. (2025). The sex hormone precursors dehydroepiandrosterone (DHEA) and its sulfate ester form (DHEAS): Molecular mechanisms and actions on human body. Int. J. Mol. Sci..

[46] Li C.W., Yu K., Shyh-Chang N., Jiang Z., Liu T., Ma S., Luo L., Guang L., Liang K., Ma W. (2022). Pathogenesis of sarcopenia and the relationship with fat mass: Descriptive review. J. Cachexia Sarcopenia Muscle.

[47] Lindqvist C., Brismar T.B., Majeed A., Wahlin S. (2019). Assessment of muscle mass depletion in chronic liver disease: Dual-energy X-ray absorptiometry compared with computed tomography. Nutrients.

