# Supplementary material for: Evaluation of Sarcopenic Obesity in Patients with MASLD
Source: Med Sci (Basel). 2026 May 15;14(2):257. doi: 10.3390/medsci14020257 (PMC13214746; doi:10.3390/medsci14020257)
Supplement: Supplementary file 1 [file medsci-14-00257-s001.zip › medsci-4271568-supplementary.pdf]

Supplementary Table S1. Medication used for AH, dyslipidemia, and T2D in our population

**Comorbidities/ Medication**	**Participants, *n* (%)**
T2D	55 (28.1)
Metformin	41 (74.5)
DPP-4 inhibitors	12 (21.8)
GLP1 receptor agonists	17 (30.9)
Sulfonylureas	1 (1.8)
SGLT2 inhibitors	13 (23.6)
Insulin	8 (14.5)
AH	72 (36.9)
ACE inhibitors/ARBs	54 (75)
Beta-blockers	29 (40.2)
Calcium channel blockers	8 (11.1)
Diuretics	3 (4.2)
Dyslipidemia	98 (49.5)
Statins	77 (78.5)
Ezetimibe	4 (4.1)

Categorical variables expressed as number of individuals *n* (percentage of the sample, %).

Abbreviations: T2D: type 2 diabetes, AH: arterial hypertension, ACE: angiotensin-converting enzyme, ARB: angiotensin receptor blocker, DPP-4: dipeptidyl peptidase-4, GLP1: glucagon-like peptide-1, SGLT2: sodium-glucose cotransporter-2

Supplementary Table S2. Comparison of patients with and without BMI-SO, by gender (univariate and multivariate analysis)

	Men				Women			
	Patients without BMI-SO (*n*=48)	Patients with BMI-SO (*n*=43)	*p*- value		Patients without BMI-SO (*n*=50)	Patients with BMI-SO (*n*=59)	*p*- value	
	**Univariate analysis**			**Multivariate analysis (OR, 95%C.I., p value)**	**Univariate analysis**			**Multivariate analysis (OR, 95%C.I., p value)**
Age, years	52.69 (± 14.64)	54.58 (± 16.62)	0.565		58.5 (± 12.39)	57.12 (± 11.05)	0.540	
Cormobidities								
T2D, *n* (%)	11 (22.9)	13 (30.2)	0.459		12 (24)	19 (32.2)	0.378	
AH, *n* (%)	12 (25)	21 (48.8)	**0.021**		15 (30)	24 (40.7)	0.314	
Dyslipidemia, *n* (%)	25 (52.1)	17 (39.5)	0.271		28 (56)	28 (47.4)	0.316	
WC, cm	102.27 (± 16.6)	115.12 (± 9.82)	**<0.001**	**1.071, 1.032-1.210, p=0.01**	94.45 (± 7.62)	108.89 (± 9.39)	**<0.001**	**1.068, 1.44-1.190, p=0.02**
BMI, kg/m²	27.31 (± 3.15)	35.11 (± 4.12)	**<0.001**		27.64 (± 2.75)	36.23 (± 4.92)	**<0.001**	

BF%	35.38 (± 5.01)	41.18 (± 4.15)	**<0.001**	44.48 (± 4.14)	50.66 (± 3.52)	**<0.001**
ALM/W, (%)	28.21 (± 2.58)	24.89 (± 2.28)	**<0.001**	23.32 (± 4.35)	19.97 (± 2.12)	**<0.001**
HbA1c (%)	5.8 (± 0.75)	5.9 (± 0.78)	0.518	5.86 (± 0.61)	6.01 (± 0.97)	0.388
Glu, mg/dl	89.91 (± 27.19)	89.67 (± 17.08)	0.963	91.91 (± 19.61)	98.88 (± 26.25)	0.140
Insulin, μU/ml	13.37 (± 10.45)	20.95 (± 13.82)	**0.007**	11.89 (± 6.16)	18.7 (± 14.51)	**0.004**
HOMA-IR	3.11 (± 2.85)	4.78 (± 3.83)	**0.029**	2.84 (± 1.86)	4.86 (± 4.9)	**0.012**
Total cholesterol, mg/dl	180.67 (± 33.34)	193.56 (± 36.18)	0.103	188.96 (± 42.56)	197.83 (± 46.39)	0.318
HDL cholesterol, mg/dl	48.77 (± 11.81)	48.01 (± 11.07)	0.770	57.18 (± 13.21)	53.19 (± 13.73)	0.138
LDL cholesterol, mg/dl	108.36 (± 28.89)	124.9 (± 32.91)	**0.024**	106.25 (± 37.29)	111.27 (± 42.35)	0.544
Triglycerides, mg/dl	123.5 (± 70.6)	128.91 (± 58.71)	0.717	117.72 (± 50.81)	136.98 (± 52.51)	0.062
AST, U/L	27.63 (± 14.72)	31 (± 16.01)	0.326	30.78 (± 24.63)	30.62 (± 25.06)	0.973
ALT, U/L	43.13 (± 47.68)	44.8 (± 28.52)	0.853	34.23 (± 29.9)	38.02 (± 36.83)	0.571
γ-GT, U/L	76.22 (± 90.55)	60.22 (± 47.47)	0.339	62.27 (± 86.77)	48.8 (± 60.11)	0.351

25(OH)VitD, ng/ml	25.02 (± 10.44)	22.77 (± 7.66)	0.292	25.01 (± 9.97)	26.35 (± 10.96)	0.528
Ferritin, ng/ml	172.14 (± 148.38)	238.45 (± 230.06)	0.120	115.6 (± 122.8)	133.74 (± 186.47)	0.572
**Hormonal status**						
FSH, mIU/mL	8.53 (± 6.59)	25.24 (± 68.25)	0.110	74.55 (± 43.08)	67.59 (±41.54)	0.421
LH, mIU/mL	4.44 (± 2.98)	7.69 (± 9)	**0.028**	23.83 (± 14.8)	22.97 (± 12.34)	0.755
DHEAS, µg/dL	200.8 (± 174.85)	178.47 (±150.84)	0.559	106.61 (± 77.9)	119.17 (± 83.27)	0.437
SHBG, nmol/L	41.08 (± 18.34)	36.03 (± 18.02)	0.224	52.18 (± 20.41)	47.54 (± 24.52)	0.314
Testosterone, ng/ml	20.3 (± 104.9)	3.77 (± 1.66)	0.355	2.01 (± 6.18)	1.34 (± 5.86)	0.590
Free testo, ng/ml	0.07 (± 0.18)	0.07 (± 0.14)	0.947	0.05 (± 0.07)	0.6 (± 3.67)	0.351
Δ4, ng/mL	0.96 (0.75)	1.05 (0.96)	0.647	0.91 (± 0.89)	0.78 (± 0.65)	0.456
**Severity of liver disease**						
FIB-4	1.11 (± 0.95)	1.13 (± 0.71)	0.911	1.3 (± 0.77)	1.21 (± 1.05)	0.638
CAP, dB/m	275.98 (± 31.84)	310.46 (± 47.15)	**<0.001**	268.12 (± 30.22)	297.21 (± 42.16)	**<0.001**

Liver stiffness, kPa	6.89 (± 4.93)	8.29 (± 7.43)	0.308		6.37 (± 3.79)	6.53 (± 3.57)	0.842	
**Functional performance tests**								
SPPB	11.49 (± 0.87)	11.03 (± 1.1)	**0.045**		11.05 (± 1.09)	10.43 (± 1.45)	**0.025**	
LFI	3.56 (± 0.64)	3.69 (± 0.49)	0.368		3.94 (± 0.52)	4.06 (± 0.53)	0.306	

Continuous variables expressed as mean (SD) and categorical variables as number of individuals *n* (percentage of the sample, %). Bold numerical values indicate statistical significance (p <0.05).

Abbreviations: T2D: type 2 diabetes, AH: arterial hypertension, BMI: body mass index, BF%: body fat percentage, ALM/W: appendicular lean mass-to-weight ratio, SO: sarcopenic obesity, WC: waist circumference, HbA1c: hemoglobin A1c, Glu: fasting glucose, HOMA-IR: homeostatic model assessment of insulin, HDL: high density lipoprotein, LDL: low-density lipoprotein, AST: aspartate aminotransferase, ALT: alanine aminotransferase, γ-GT: gamma-glutamyl transferase, 25(OH)VitD: 25-hydroxy-vitamin D, FSH: follicle-stimulating hormone, LH: luteinizing hormone, DHEAS: dehydroepiandrosterone sulfate, SHBG: sex hormone-binding globulin, Testo: testosterone, Δ4: Δ4 androstenedione, FIB-4: fibrosis-4 index, CAP: controlled attenuation parameter, SPPB: short physical performance battery, LFI: liver frailty index.

Supplementary Table S3. Comparison of patients with and without SO - based on AIMSO score

	**Without SO *n* = 131 (65.5%)**	**With SO *n* = 69 (34.5%)**	***p*-value**
Age, years	55.77 (± 13.13)	56 (± 14.67)	0.909
Sex, male, *n* (%)	24 (18.3)	67 (97.1)	**<0.001**
Comorbidities			
T2D, *n* (%)	34 (25.9)	21 (30.4)	0.404
AH, *n* (%)	41 (31.3)	31 (44.9)	**0.05**
Dyslipidemia, *n* (%)	63 (48.1)	35 (50.7)	0.688
WC, cm	100 (92 - 104)	111 (98 - 121)	**<0.001**
BMI, kg/m²	30 (± 5.1)	34.6 (± 5.47)	**<0.001**
BF%	39.7 (36.12 - 46.45)	50.3 (42.5 - 53.12)	**<0.001**
ALM/W, (%)	25.21 (21.68 - 28.04)	21.15 (19.77 - 24.02)	**<0.001**
HbA1c (%)	5.7 (5.3 - 6.1)	5.8 (5.4 - 5.9)	0.375
Glu, mg/dl	87 (79 - 95)	87 (82 - 101)	0.562
Insulin, µU/ml	13.3 (8.03 - 19.9)	13.5 (8.7 - 17.2)	0.981
HOMA-IR	3.09 (1.66 - 4.42)	3 (1.7 - 4.13)	0.872
Total cholesterol, mg/dl	186.12 (± 40.15)	198.24 (± 41.21)	0.053
HDL cholesterol, mg/dl	52.3 (± 13.36)	51.81 (± 12.63)	0.806
LDL cholesterol, mg/dl	107.58 (± 36.68)	119.47 (± 35.12)	**0.043**
Triglycerides, mg/dl	124.3 (± 61.35)	132.55 (± 52.55)	0.357
AST, U/L	23 (20 - 29)	22 (17 - 30)	0.14
ALT, U/L	25 (22 - 47)	23 (15 - 32)	0.12
γ-GT, U/L	36 (20 - 95)	28 (15 - 42)	**0.027**
25(OH)VitD, ng/ml	25.06 (± 10.12)	24.84 (± 9.85)	0.888
Ferritin, ng/ml	128 (80.5 - 209)	86.9 (65.3 - 155)	0.192
**Hormonal status**			
FSH, mIU/ml	21.4 (7.26 - 78.8)	41.1 (9.83 - 87.9)	0.131

LH, mIU/ml	12.02 (3.61 - 25.3)	10.7 (4.31 - 30.4)	0.109
DHEAS, µg/dl	115 (72.8 - 206)	102 (61.3 - 176)	0.610
SHBG, nmol/L	47.1 (30.8 - 60.3)	42.1 (26.1 – 53.3)	0.060
Testo, ng/ml	0.48 (0.19 – 3.98)	1.43 (0.14 - 4.41)	0.208
Free Testo, ng/ml	0.006 (0.002 - 0.079)	0.025 (0.003 - 0.073)	0.969
Δ4, ng/ml	0.7 (0.39 - 1.34)	0.7 (0.3 – 0.9)	0.207
**Severity of liver disease**			
FIB-4	1.1 (0.83 - 1.57)	1.04 (0.72 - 1.2)	0.11
CAP, dB/m	274 (251 - 284)	289 (263 - 330)	**0.044**
Liver stiffness, kPa	5.7 (5.11 - 6.9)	5.6 (4.7 - 6.3)	0.15
**Functional performance tests**			
SPPB	11 (11 - 12)	11.5 (10 - 12)	0.143
LFI	3.81 (3.42 - 4.18)	3.95 (3.5 - 4.36)	**0.043**

Continuous variables expressed as mean (SD) or median with interquartile range (IQR: 25th - 75th percentile) and categorical variables as number of individuals *n* (percentage of the sample, %). Bold numerical values indicate statistical significance ($p < 0.05$).

Abbreviations: T2D: type 2 diabetes, AH: arterial hypertension, BMI: body mass index, BF%: body fat percentage, ALM/W: appendicular lean mass-to-weight ratio, SO: sarcopenic obesity, WC: waist circumference, HbA1c: hemoglobin A1c, Glu: fasting glucose, HOMA-IR: homeostatic model assessment of insulin, HDL: high density lipoprotein, LDL: low-density lipoprotein, AST: aspartate aminotransferase, ALT: alanine aminotransferase, γ-GT: gamma-glutamyl transferase, 25(OH)VitD: 25-hydroxy-vitamin D, FSH: follicle-stimulating hormone, LH: luteinizing hormone, DHEAS: dehydroepiandrosterone sulfate, SHBG: sex hormone-binding globulin, Testo: testosterone, Δ4: Δ4 androstenedione, FIB-4: fibrosis-4 index, CAP: controlled attenuation parameter, SPPB: short physical performance battery, LFI: liver frailty index.
